# Dynamics of the Humoral Immune Response to a Prime-Boost Ebola Vaccine: Quantification and Sources of Variation

**DOI:** 10.1128/JVI.00579-19

**Published:** 2019-08-28

**Authors:** Chloé Pasin, Irene Balelli, Thierry Van Effelterre, Viki Bockstal, Laura Solforosi, Mélanie Prague, Macaya Douoguih, Rodolphe Thiébaut

**Affiliations:** a INSERM U1219, University of Bordeaux, Bordeaux, France; b INRIA SISTM team, Talence, France; c Vaccine Research Institute, Créteil, France; d Janssen Pharmaceutica N. V., Beerse, Belgium; e Janssen Vaccines & Prevention B. V., Leiden, The Netherlands; University of North Carolina at Chapel Hill

**Keywords:** antibody response, Ebola, mechanistic modeling, vaccine

## Abstract

The Ebola vaccine based on Ad26.ZEBOV/MVA-BN-Filo prime-boost regimens is being evaluated in multiple clinical trials. The long-term immune response to the vaccine is unknown, including factors associated with the response and variability around the response. We analyzed data from three phase 1 trials performed by the EBOVAC1 Consortium in four countries: the United Kingdom, Kenya, Tanzania, and Uganda. Participants were randomized into four groups based on the interval between prime and boost immunizations (28 or 56 days) and the sequence in which Ad26.ZEBOV and MVA-BN-Filo were administered. Consecutive enzyme-linked immunosorbent assay (ELISA) measurements of the IgG binding antibody concentrations against the Kikwit glycoprotein (GP) were available for 177 participants to assess the humoral immune response up to 1 year postprime. Using a mathematical model for the dynamics of the humoral response, from 7 days after the boost immunization up to 1 year after the prime immunization, we estimated the durability of the antibody response and the influence of different factors on the dynamics of the humoral response. Ordinary differential equations (ODEs) described the dynamics of antibody response and two populations of antibody-secreting cells (ASCs), short-lived (SL) and long-lived (LL). Parameters of the ODEs were estimated using a population approach. We estimated that half of the LL ASCs could persist for at least 5 years. The vaccine regimen significantly affected the SL ASCs and the antibody peak but not the long-term response. The LL ASC compartment dynamics differed significantly by geographic regions analyzed, with a higher long-term antibody persistence in European subjects. These differences could not be explained by the observed differences in cellular immune response.

**IMPORTANCE** With no available licensed vaccines or therapies, the West African Ebola virus disease epidemic of 2014 to 2016 caused 11,310 deaths. Following this outbreak, the development of vaccines has been accelerated. Combining different vector-based vaccines as heterologous regimens could induce a durable immune response, assessed through antibody concentrations. Based on data from phase 1 trials in East Africa and Europe, the dynamics of the humoral immune response from 7 days after the boost immunization onwards were modeled to estimate the durability of the response and understand its variability. Antibody production is maintained by a population of long-lived cells. Estimation suggests that half of these cells can persist for at least 5 years in humans. Differences in prime-boost vaccine regimens affect only the short-term immune response. Geographical differences in long-lived cell dynamics were inferred, with higher long-term antibody concentrations induced in European participants.

## Abstract (precis)

With no available licensed vaccines or therapies, the West African Ebola virus disease epidemic of 2014 to 2016 caused 11,310 deaths. Following this outbreak, the development of vaccines has been accelerated. Combining different vector-based vaccines as heterologous regimens could induce a durable immune response, assessed through antibody concentrations. Based on data from phase 1 trials in East Africa and Europe, the dynamics of the humoral immune response from 7 days after the boost immunization onwards were modeled to estimate the durability of the response and understand its variability. Antibody production is maintained by a population of long-lived cells. Estimation suggests that half of these cells can persist for at least 5 years in humans. Differences in prime-boost vaccine regimens affect only the short-term immune response. Geographical differences in long-lived cell dynamics were inferred, with higher long-term antibody concentrations induced in European participants.

## INTRODUCTION

Since the 2014–2016 outbreak of Ebola virus (EBOV) disease (EVD) in West Africa that caused 28,616 cases and 11,310 fatalities (1), the clinical development of several Ebola vaccine candidates has been accelerated. Among the vaccine candidates, a heterologous prime-boost strategy combining immunizations with Ad26.ZEBOV (Janssen Vaccines and Prevention) and MVA-BN-Filo (Bavarian Nordic) is being developed by Janssen (2, 3). Prime-boost regimens are expected to be more immunogenic than prime-only vaccination strategies (4–7). In nonhuman primate studies, heterologous prime-boost filovirus vaccination regimens elicited an immune response able to protect vaccinated animals against lethal Ebola virus challenge (8). Different immunization regimens using Janssen’s vaccine candidate have been evaluated in clinical trials. In particular, we focus here on three phase 1 trials performed by the EBOVAC1 Consortium on healthy adult volunteers in four countries: the United Kingdom (2, 9), Kenya (10), and Uganda and Tanzania (11). The consortium is part of the Innovative Medicines Initiative Ebola+ program (12), which aims to assess a novel prime-boost preventive vaccine regimen against EVD. Results of the three phase 1 trials showed no vaccine-related serious adverse events and persistent levels of IgG binding antibodies in all vaccine recipients.

One of the potential assets of the Ad26.ZEBOV/MVA-BN-Filo vaccine regimens is the establishment of a long-term immune response, which is in part characterized by the Ebola virus glycoprotein (GP)-specific binding antibody response after vaccination. Although no immune correlate of protection has been identified yet, preclinical studies have shown that the peak antibody concentrations postvaccination are correlated with survival after intramuscular challenge in a nonhuman primate model, which is the closest model to humans (13–15). Whether circulating antibody concentrations also correlate with long-term protection is not established; however, it is of particular interest to quantify the dynamics of the humoral immune response and to estimate the durability of the antibody response. We proposed to use a mathematical model to address these questions. We had a unique opportunity to analyze the data from the three trials in the context of EBOVAC1, because they were conducted almost simultaneously with very similar study protocols. The uniqueness of the data also relied on the large number of consecutive immunogenicity measurements following the boost immunization.

Most of the models that were already developed for the dynamics of the antibody response focused on the decline of the antibody concentrations after the peak response. Linear or piecewise-linear decreases of the antibody response were fitted to data from a large number of vaccines, including hepatitis B vaccine (16), combined diphtheria, tetanus, and pertussis vaccine (17, 18), Japanese encephalitis chimeric virus vaccine (19), hepatitis A vaccine (20, 21), and human papillomavirus 16/18 vaccine (22–24). However, linear mixed models are limited in term of biological interpretation. Conversely, the structure of mechanistic models is based on biology and is able to capture nonlinear interactions. The estimation of the model parameters gives a quantification of the biological phenomenon. Only a few within-host models were developed to describe the humoral immune response following vaccination. The dynamics of antibody-secreting cells (ASCs) after vaccinia virus vaccination of human volunteers were described (25) by extending a widely known model for the CD8 T cell response (26, 27). However, this model did not account for the immunologic hypothesis that antibodies are produced by several populations of ASCs. Indeed, it has been suggested that the vast majority of plasma cells generated through immunization are short-lived (SL) cells (28, 29), peaking 7 days after the immunization and lasting very shortly in the organism (29–32). However, the half-life of antibodies was estimated at between 20 and 50 days in several studies (33–39). Therefore, the persistence of antibody response, observed to last for several years (40), is expected to be generated by long-lived (LL) plasma cells (29, 41–44). Using long-term data following hepatitis A vaccination (up to 10 years after the boost immunization), an ordinary differential equation (ODE)-based mechanistic model helped quantify 3 scales of the humoral response dynamics (45), corresponding to the life spans of antibodies (around 20 to 30 days), and two populations of ASCs (one living several months and the other 1 decades). In this study, we used the same mechanistic model for the humoral immune response, with two populations of ASCs (SL and LL) and the antibody population. Parameters were estimated on data available from three trials of the EBOVAC1 Consortium, with a 1-year follow-up for participants of the study including up to 9 consecutive measurements of antibody concentrations. This model allowed us to quantify the dynamics of the humoral immune response following different prime-boost vaccine regimens.

## RESULTS

### Mechanistic model of the immune response.

**FIG 1:**
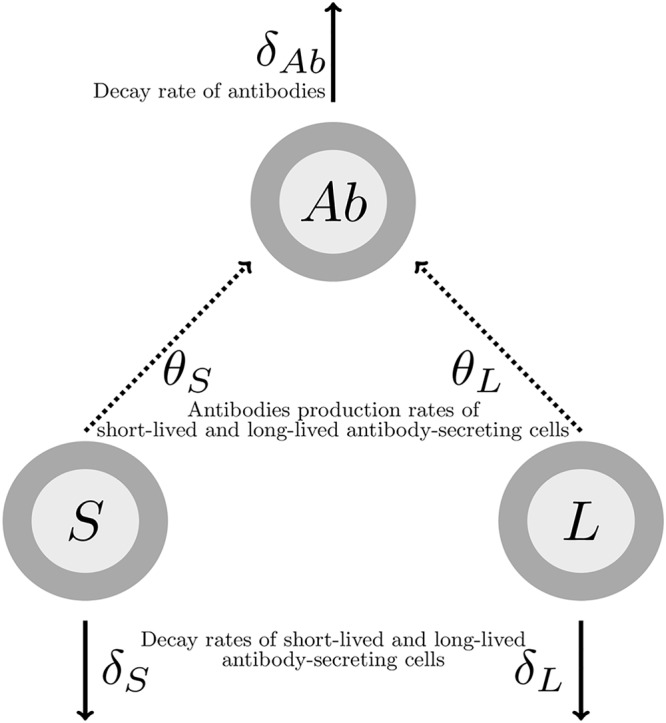
Model of the humoral immune response from 7 days after the boost immunization. *S*, SL ASCs; *L*, LL ASCs; *Ab*, antibodies.

A preliminary analysis was performed to estimate linear trends of the antibody concentration decrease from 21 days after the boost immunization onwards. The method and results of this analysis are detailed in the appendix. This analysis showed in particular the need to model two phases of antibody decline. A mechanistic model was used to fit these dynamics. Based on previous work in immunology (46) and modeling (25, 45), we made the hypothesis that antibodies are produced by two distinct populations of ASCs, which can be distinguished by two different half-lives: some are assumed SL and others LL. We made the assumption that from 7 days after the boost immunization, both populations of cells decay with time. This decay is applied to the whole compartment of cells and could mean either that these cells are still generated but their death rate is higher than their proliferation rate or that cells are not generated anymore; in this case, the decay corresponds to their net loss. In any case, the assumption can be justified by some experimental evidence that ASCs peak a few days after reaction to pathogen and decrease thereafter (14, 31, 32, 47). A recent review also suggested that the kinetics of ASCs were similar among different pathogens, with a peak response around 7 or 8 days following infection (48). As ASCs peak around 7 days after immunization, it could then reasonably be assumed that they decay after this time. LL cells are expected to play a role on a longer time scale, as these cells are the ones supposed to sustain antibodies (43, 44). The model of the dynamics of the humoral immune response from 7 days after boost immunization and its parameters are represented in Fig. 1. SL and LL ASCs decay, respectively, at rates δ*_S_* and δ*_L_* and produce antibodies at rates θ*_S_* and θ*_L_*. Antibodies decay at rate δ*_Ab_*. SL and LL values 7 days after the boost immunization are unknown and written, respectively, as *S*_0_ and *L*_0_. For identifiability issues, we defined two new parameters: φ*_S_* = θ*_S_S*_0_ and φ*_L_* = θ*_L_L*_0_. They correspond to the rate of antibody production times the ASC baseline level, which we call the influx. We used a population approach to estimate parameters δ*_Ab_*, δ*_S_*, δ*_L_*, φ*_S_*, and φ*_L_* and to assess the effects of the different factors on these parameters and also their interindividual variability (see equation 5 in Materials and Methods).

### Descriptive analysis of the data.

**FIG 2:**
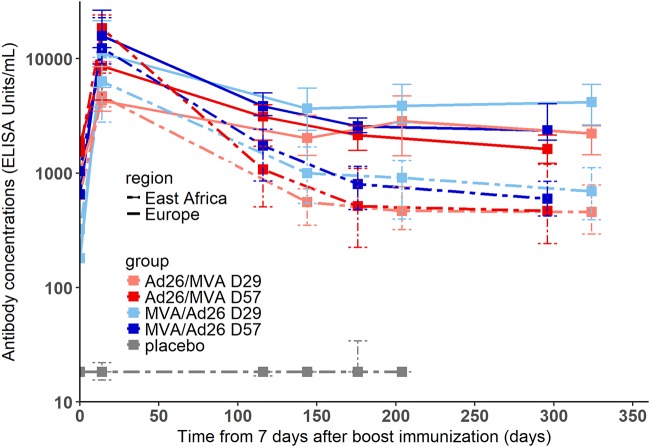
Dynamics of antibody concentrations (in log_10_ scale) from 7 days postboost in European and East African subjects of each group of vaccination. Each color corresponds to a vaccination group, as shown in the key. Solid lines correspond to medians in European subjects and dashed lines to medians in East African subjects. The 25th to 75th quantiles are also represented.

**FIG 3:**
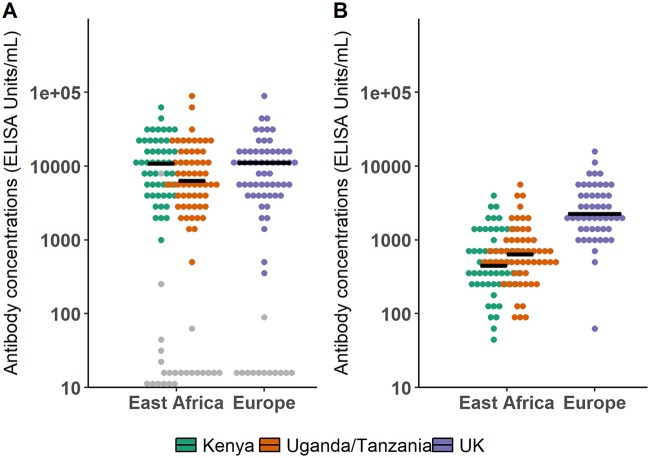
Comparison of antibody concentrations (in log_10_ scale) in European and East African subjects. Horizontal lines correspond to median values within each study. Gray points correspond to placebo recipients. (A) Box plot of antibody concentrations at time of the observed peak (21 days after the boost immunization) in European and East African subjects. (B) Box plot of antibody concentrations 1 year after the prime immunization in European and East African subjects.

A summary of the characteristics of the data set is given in Table 1. As shown in Fig. 2, dynamics of antibodies from 7 days after the boost immunization were similar across all groups and geographic regions: a peak of the antibody response was observed 21 days after the boost immunization, and then antibody concentrations followed a biphasic decay with a first sharp decrease followed by a slower decline. Antibody concentrations at specific time points are detailed in Table 1 and Fig. 3. There was no statistical difference in antibody concentrations at the peak of the response between European and East African subjects (*P* value of *t* test on log_10_-transformed antibody concentrations = 0.76). However, 1 year after the prime immunization, the antibody concentrations of European subjects were statistically significantly higher than those of East African subjects (*P* value of *t* test on log_10_-transformed antibody concentrations < 3.10^−15^), the European mean value being 23% higher than the East African one.

**TABLE 1:** Summary of data characteristics

Parameter	Value for group			
	Europe, UK	East Africa		Total
		Kenya	Uganda/Tanzania	
Participants, no.	59	59	59	177
Group MVA/Ad26 D29	15	14 (1 non completed)	15	44
Group MVA/Ad26 D57	15	15	14 (1 noncompleted)	44
Group Ad26/MVA D29	15	15	15	45
Group Ad26/MVA D57	14 (1 lost to follow-up)	15	15	44
Sex, no. (%)				
Men	21 (36)	42 (71)	47 (80)	110 (62)
Women	38 (64)	17 (29)	12 (20)	67 (38)
Age, in yrs, mean (SD)	35.5 (9.9)	25.9 (6.2)	26.4 (6.5)	29.7 (9.1)
BMI, in kg/m^2^, mean (SD)	25.2 (4.0)	23.1 (3.6)	22.3 (3.7)	23.6 (4.0)
Antibody concn, in log_10_ ELISA units/ml, mean (SD), and no. of participants				
7 days postboost	2.83 (0.69), 59	3.01 (0.65), 58	2.75 (0.71), 59	2.86 (0.69), 176
21 days postboost (peak)	3.95 (0.43), 59	4.01 (0.39), 59	3.85 (0.43), 59	3.94 (0.42), 177
1 yr postprime	3.38 (0.40), 51	2.70 (0.43), 58	2.79 (0.39), 59	2.94 (0.50), 168

**FIG 4:**
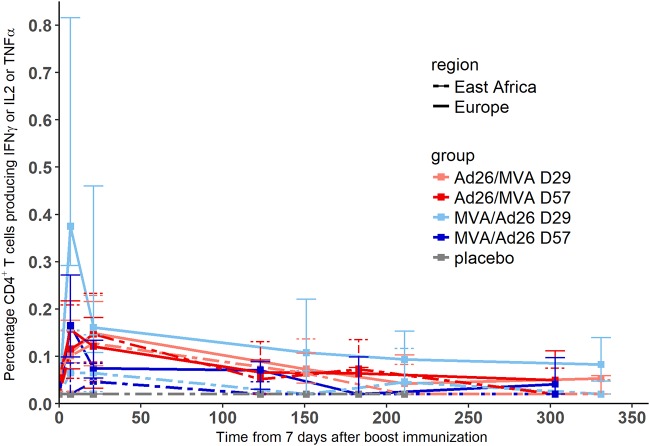
Dynamics of percentages of CD4^+^ T cells producing at least one of the three cytokines IL-2, IFN-γ, and TNF-α from the time of boost immunization. The lower limit of quantification was 0.04, and values under this limit were imputed to half of it (=0.02). Each color corresponds to a vaccination group, as shown in the color key. Solid lines correspond to medians in European subjects and dashed lines to medians in East African subjects. The 25th to 75th quantiles are also represented.

**FIG 5:**
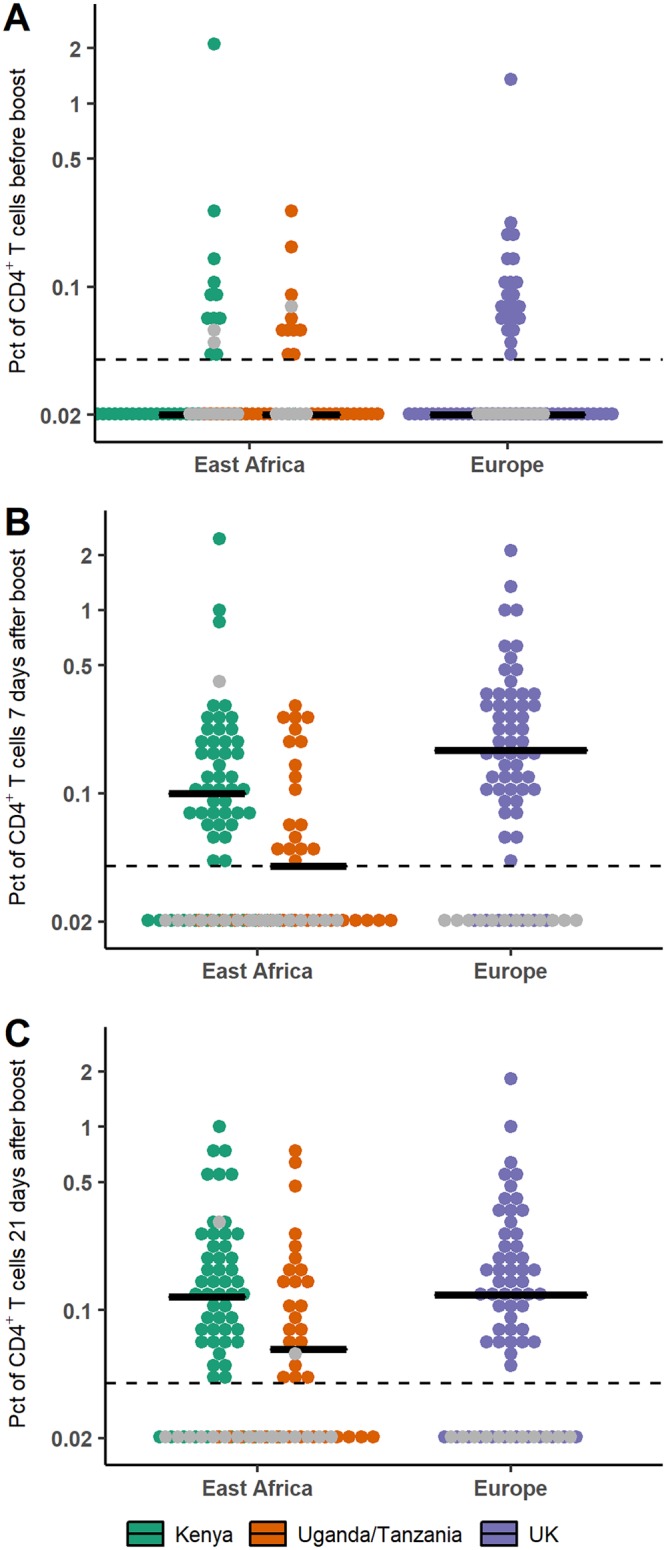
Comparison of percentages (Pct; in log scale) of CD4^+^ T cells producing at least one of the three cytokines IL-2, IFN-γ, and TNF-α in European and East African subjects after prime prior to boost immunization (A), 7 days after boost immunization (B), and 21 days after boost immunization (C). Horizontal lines correspond to the median value within each trial. Dashed lines correspond to the lower limit of quantification (=0.04); values under this limit were imputed to half of it (=0.02). Gray points correspond to placebo recipients.

The cellular response was also studied from the time of the boost immunization onwards. In particular, we focused on the total percentage of stimulated CD4^+^ T cells producing at least one of the three cytokines interleukin-2 (IL-2), gamma interferon (IFN-γ), and tumor necrosis factor alpha (TNF-α). The dynamics of these cytokine-secreting CD4^+^ T cells after the boost immunization are shown in Fig. 4. Dynamics were very similar across all groups, with a peak response measured between 7 and 21 days after the boost immunization. We plotted in Fig. 5 the distributions of the percentages of CD4^+^ T cells in European and East African subjects at specific time points and tested if there was a difference with a two-sided Wilcoxon rank sum test. There was a significant difference between European and East African subjects after prime prior to boost immunization and 7 days after the boost immunization (*P* value = 0.015 and *P* value < 0.001, respectively), with higher percentages of CD4^+^ T cells in European than in East African subjects. At 21 days after the boost immunization, this difference was no longer significant (*P* value = 0.23).

### Parameter estimation and goodness of fit of the model.

**FIG 6:**
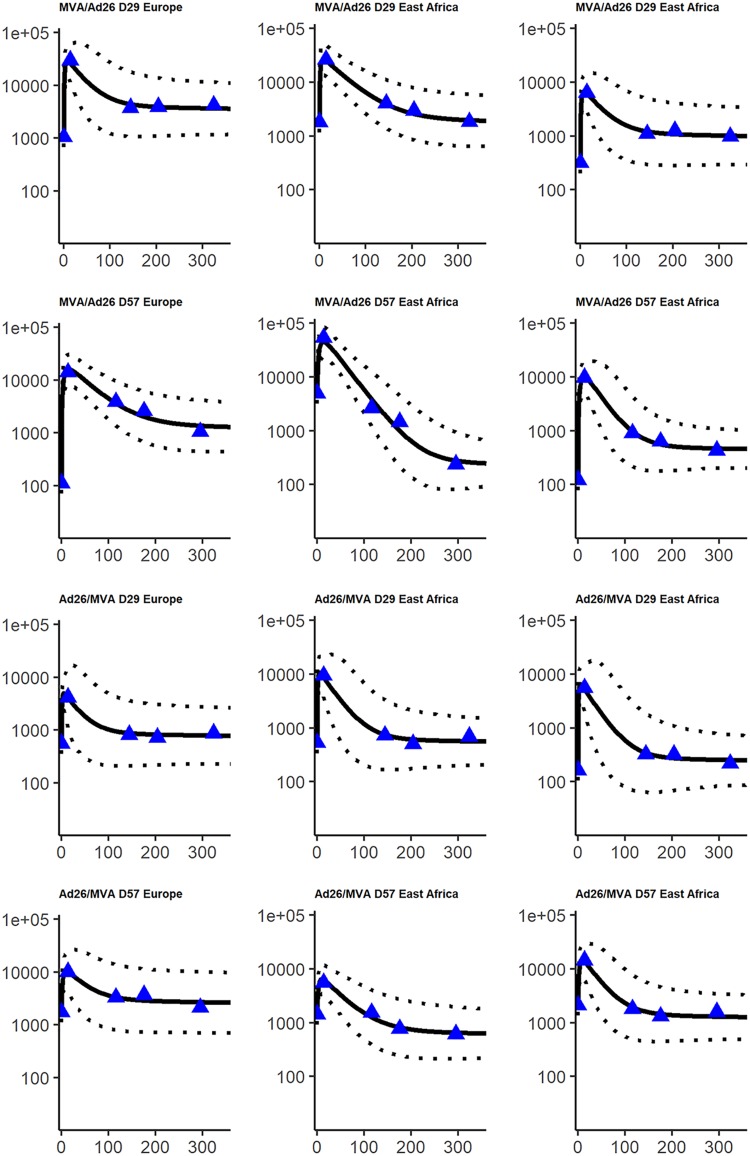
Fits of a random sample of subjects. The *x* axis corresponds to the time from 7 days after boost immunization (in days), and the *y* axis corresponds to the antibody concentrations (in ELISA units/milliliter; log_10_ scale). For each subject, blue triangles correspond to the observed data, the solid line corresponds to the prediction from the model, and the dashed line corresponds to the 95% prediction interval, accounting for the uncertainty on parameter estimation and the measurement error.

**FIG 7:**
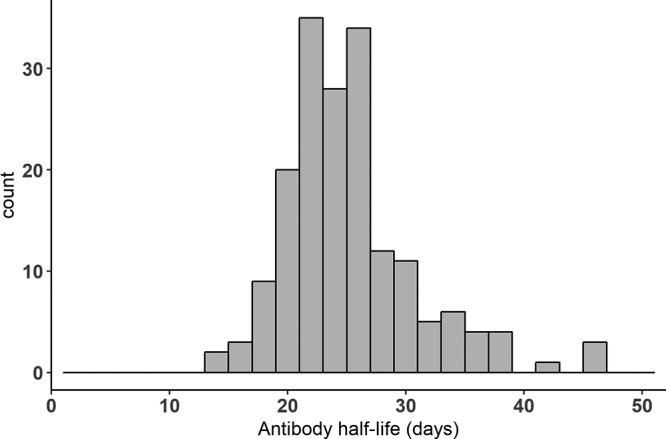
Histogram of estimated antibody half-life in all participants.

**FIG 8:**
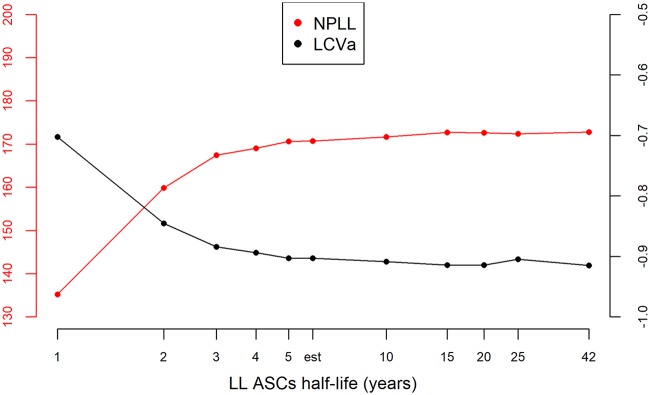
Profile likelihood on parameter δ*_L_.* The left axis corresponds to the nonpenalized log likelihood in red (NPLL), which needs to be maximized. The right axis corresponds to the LCVa in black, which needs to be minimized. Both criteria are computed for several values of long-lived cell half-life, including the estimated (est) one, and represented on a log-scaled axis.

Using a population approach, we estimated the value of the parameters of the model and assessed the effect of different factors on these parameters. Prior distributions were used to constrain the parameter values, according to previous biological knowledge. This is detailed in Materials and Methods. After selection of the model, we found that the vaccine regimens induced different mean decay rates of SL ASCs (δ*_S_*). We also estimated different mean values of the LL influx parameter (φ*_L_* = *θ_L_L*_0_) between East African and European subjects. The variation of three parameters (both influx φ*_S_* and φ*_L_* and the decay rate of antibodies δ*_Ab_*) could not be fully explained by the measured factors (geographic region and vaccine regimen) and was handled with normal random effects. This model allowed to fit well the data of participants, as shown in Fig. 6. Parameters estimation is displayed in Table 2 and allowed quantification of the humoral immune response to the prime-boost regimen. The mean value for the half-life of circulating antibodies across participants was estimated at 24 days (95% confidence interval, 22, 26), and 5th to 95th quantiles of the distribution of individual values ranged from 18 to 36 days. The histogram of the estimated antibody half-life in all participants is shown in Fig. 7. This estimation was lower than values estimated through passive immunity (37–39) but still seemed biologically plausible and consistent with some previous studies (35, 46). Sensitivity analyses were realized on the estimation of that parameter: in particular, we estimated the parameters by using a more constraining prior distribution on the decay rate of antibodies δ*_Ab_*, corresponding to a mean half-life value of 42 days, with 5th to 95th quantiles of the parameter distribution at 34 to 51 days. Compared to the previous estimation of a 24-day half-life, the new estimate of the antibody half-life with the stronger prior barely changed (27 days [95% confidence interval, 25, 29]), showing robustness in the estimation of this parameter. Moreover, estimation of ASC half-life allowed us to distinguish two populations of ASCs, one with a very short half-life (only a few days) and one with a much longer half-life (a few years). The difference in value between φ*_S_* and φ*_L_*, with φ*_S_* > φ*_L_*, also suggested that SL cells were present at a much higher level 7 days after the boost immunization and/or they produced many more antibodies than LL cells. These estimations supported the hypothesis of the generation of a very quickly reactive population of cells with a short life span and long-term antibody production sustained by another population of cells able to last several years in the organism (29). We estimated the half-life of these LL cells to be 6.0 years (95% confidence interval, 2.7, 13). The upper bound of the confidence interval is actually artificially introduced by the normal approximation of the parameter distribution, but we did not have enough information to determine with precision the upper bound. Indeed, data were only available up to 1 year after the prime immunization, and the decline of this population is slow. We performed a profile likelihood to explore the identifiability of parameter δ*_L_*, as shown in Fig. 8. For several values of δ*_L_*, the model was estimated and model criteria were computed (nonpenalized log likelihood and likelihood cross-validation criteria [LCVa] [49]): the resulting profile showed a flat behavior after a half-life value of 5 years, meaning that data up to 1 year did not allow us to distinguish between an estimated half-life of 5 years or more. The estimation based on the currently available data suggested that half of the long-lived ASCs generated at 7 days after the boost immunization would persist for at least 5 years.

**TABLE 2:** Parameter estimation

Parameter	Mean	95% confidence interval
Antibody half-life (days), log_2_/δ_Ab_	24	22, 26
Long-lived cell half-life (yrs), log_2_/δ*_L_*	6.0	2.7, 13
Short-lived cell half-life (days), log_2_/δ*_S_*		
MVA/Ad26 D29 group	2.0	1.3, 3.0
MVA/Ad26 D57 group	4.9	3.1, 7.7
Ad26/MVA D27 group	1.2	0.8, 1.9
Ad26/MVA D57 group	3.0	1.9, 4.7
ϕ*_S_* (ELISA units/ml days^−1^)	2,755	1,852, 4,100
ϕ*_L_* (ELISA units/ml days^−1^)		
East African participants	16.6	13.7, 20.1
European participants	70.7	54.0, 92.7
σ_ϕ*_S_*_ (interindividual SD on ϕ*_S_*)	0.92	0.83, 1.01
σ_ϕ*_L_*_ (interindividual SD on ϕ*_L_*)	0.85	0.78, 0.92
σ_δ*_Ab_*_ (interindividual SD on δ*_Ab_*)	0.30	0.24, 0.36
σ*_Ab_* (SD on observations)	0.10	0.10, 0.10

### Factors influencing the dynamics of the humoral response.

**FIG 9:**
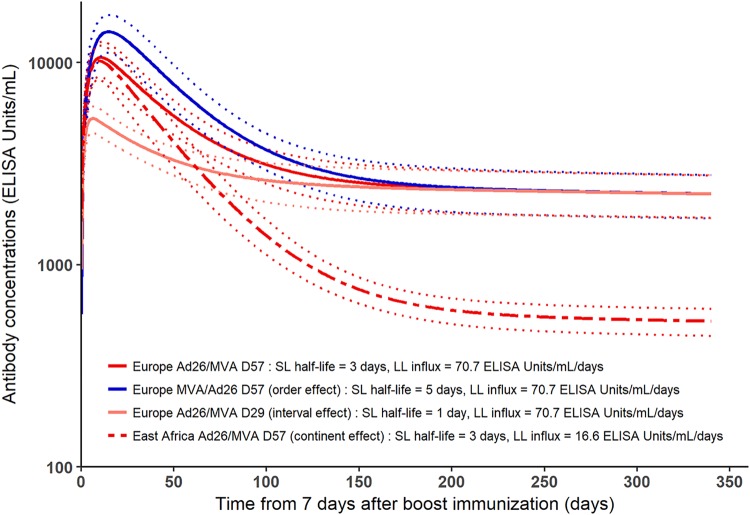
Marginal predictions of antibody concentration dynamics from the model (in log_10_ scale). Marginal predictions show the effect of covariates on antibody concentration dynamics. The dark red plain line (Europe Ad26/MVA D57 group) differs from the dark blue plain line (Europe MVA/Ad26 D57) only by order of immunizations, showing that MVA/Ad26 order implies a higher peak of antibody concentrations than the Ad26/MVA order. The dark red plain line (Europe Ad26/MVA D57 group) differs from light red plain line (Europe Ad26/MVA D29) only by interval between prime and boost immunizations, showing that a boost at day 56 induces a higher peak of antibody concentrations than a boost at day 28. Finally, the dark red plain line (Europe Ad26/MVA D57 group) differs from the dark red dashed line (East Africa Ad26/MVA D57 group) only by geographic region, showing that European subjects have a similar antibody peak as East African ones but higher sustained antibody concentrations. For all curves, light dashed lines correspond to 95% confidence intervals accounting for the uncertainty on parameters estimation.

Table 3 summarizes the effect of covariates on the biological parameters of the model. As detailed in Table 2, we estimated that the mean SL ASCs half-life varies from 1.2 days to 4.9 days, depending on the vaccine regimen. This estimation was consistent with findings on the kinetics of ASCs following infection/vaccination, suggesting that circulating plasmablasts peak at 7 days after boost immunization but are absent after 10 to 14 days (48). The model estimates suggested that Ad26/MVA induced a shorter half-life than did MVA/Ad26 and boost immunization at day 56 induced a longer half-life than did boost immunization at day 28. These differences had an impact on the early antibody dynamics: the longer the half-life of SL ASCs, the higher the peak of antibody concentrations. The impact of the value of the SL ASCs half-life was negligible in the longer term, as shown in Fig. 9. This suggests that the different prime-boost regimens induced different early responses after the boost immunization but no difference in the duration of the antibody responses.

**TABLE 3:** Effects of covariates

Covariate	Parameter				
	ϕ*_S_*	δ*_S_*	ϕ*_L_*	δ*_L_*	δ*_Ab_*
Order of administration		δ_*S*_ (Ad26/MVA) > δ_*S*_ (MVA/Ad26)			
Interval between immunizations		δ_*S*_ (D57) < δ_*S*_ (D29)			
Geographic region			ϕ*_L_*(Europe) > ϕ_*L*_(East Africa)		

Descriptive analysis of the data suggested a significant difference of mean antibody concentrations 1 year after the prime immunization between the East African and European trials. Estimation of the model helped in understanding and quantifying this difference. We found a significant different value of parameter φ*_L_* between East African and European subjects. This difference in φ*_L_* value may explain why European subjects reached higher antibody concentrations 1 year after the immunization than East African subjects, as shown in Fig. 9. Despite different antibody concentrations reached 1 year after the prime immunization in the European and East African populations, antibody concentrations are expected to decline at the same pace: indeed, the value of δ*_L_* was not estimated to vary between these subjects, and the decay of antibodies at longer term is expected to be driven by the decay of LL ASCs. In particular, we estimated a mean decrease of the log_10_ antibody concentrations of 3% between 1 and 2 years after the prime immunization in the population of all subjects from the three phase 1 studies used to estimate the model parameters. Altogether, the model estimations of φ*_L_* and δ*_L_* suggested that the LL ASCs are able to persist as long in European subjects as in East African ones but are produced in a higher number after the boost immunization and/or secrete more antibodies in European subjects than in East African ones. This difference could result from a more activated immune environment at baseline in East African subjects (50). From this hypothesis, as environmental characteristics differ between Kenya and Uganda/Tanzania, we could expect more interindividual variability of the value of φ*_L_* within the East African group of participants compared to European group. A Fisher test for equality of variances showed that there was no significant difference of variance for φ*_L_* between East African and European values (*P* value = 0.30), meaning that there was no additional unexplained variability in the East African group compared to the European one.

**FIG 10:**
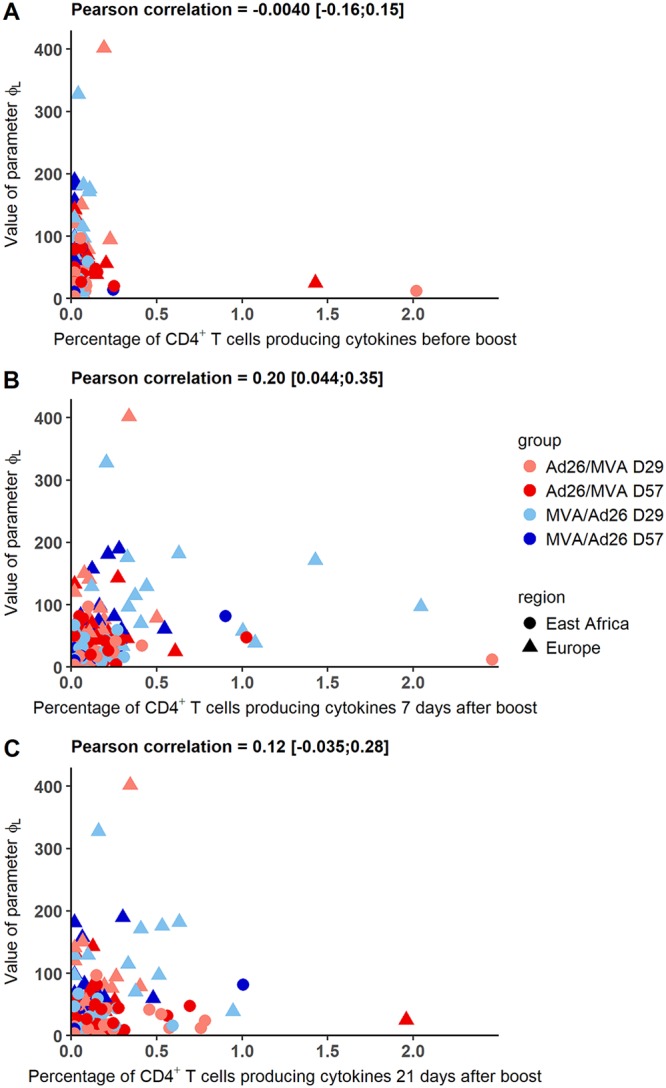
Value of parameter φ*_L_* versus the percentage of CD4^+^ T cells producing IL-2, IFN-γ, or TNF-α after prime prior to boost, 7 days after the boost immunization, and 21 days after the boost immunization in European and East African subjects. Each color corresponds to a vaccination group as shown in the key.

Following this result, we explored if the estimated difference between East African and European subjects could be explained by the magnitude of the cellular CD4^+^ T cell response. It came from the hypothesis that differences in the pathogens to which individuals are exposed during everyday life could have an effect on the cellular response (51). As CD4^+^ T cells are required for the humoral immune response, we made the hypothesis that the difference between East African and European subjects could be mediated by a difference in the T helper responses early after the boost immunization. In the mechanistic model, the difference was estimated on parameter φ*_L_*: the mean value over European subjects was higher than in East African subjects. As there was also a random effect on φ*_L_*, we were able to compute the individual estimated value of this parameter. We computed the correlation between the value of φ*_L_* and the percentage of CD4^+^ T cells producing at least one of the three cytokines IL-2, IFN-γ, and TNF-α at different time points: after prime prior to boost immunization and 7 and 21 days after the boost immunization. Results are displayed in Fig. 10. We did not observe any clear relationship between the CD4^+^ T cell percentages and φ*_L_* values. Pearson correlation coefficients were only significantly different from 0 at 7 days after boost immunization, with a moderate value of 0.2. To further explore the hypothesis that the difference of value of φ*_L_* could be mediated by the T helper response, we introduced the percentage of the CD4^+^ T cells producing cytokines 7 days after the boost immunization in the mechanistic model as a covariate on φ*_L_*. Effect of the covariate was added and tested separately on φ*_L_*, with or without the geographic region variable, as shown in equation 6 in Materials and Methods. Without the geographic region variable, the estimated effect of the CD4^+^ T cell was significant (*P* value = 0.03), but the likelihood of the model was much lower than for the model including the geographic region variable without the CD4 variable (136.34 versus 171.97). In a model including both geographic region and CD4 variables, the estimated effect of the CD4^+^ T cell response was not significant (*P* value = 0.64). Overall, these results suggested that the difference of φ*_L_* value between the geographic regions could not be explained by the measure of the percentage of CD4^+^ T cells producing at least one of the cytokines IL-2, IFN-γ, and TNF-α 7 days after the boost immunization.

## DISCUSSION

The mechanistic model accounting for two populations of ASCs allowed us to quantify the dynamics of the antibody response following different prime-boost vaccine regimens. In particular, it allowed us to estimate a lower bound of the durability of the antibody response through LL plasma cells. Moreover, we were able to identify several factors influencing the response to vaccine. We found that vaccine regimen impacts the magnitude of the early antibody response through the dynamics of the SL ASCs but has no effect on the LL ASCs and thus on the long-term persistence of antibodies. It suggests a minor impact of the interval between the prime and the boost immunizations on the long-term level of the binding antibodies.

The dynamics of LL ASCs were estimated to differ by geographic region, inducing a higher long-term level of antibodies in European subjects than in East African ones. Several factors could contribute to the geographic effect, such as HLA subtypes, nutritional status, coinfections, or preexisting immunity. Demographic factors could also play a role in this difference, although no significative effect of sex and age was found on the decrease of the antibody concentrations in the linear mixed model or on the parameter φ*_L_* (see appendix for details). The absence of association between this difference and circulating CD4^+^ T cells producing cytokines does not exclude alternative effects of the CD4^+^ T cells on the humoral response, for example, a link with plasma cells and antibody production at the level of the lymphoid organs. The difference of immune responses between different geographic regions has already been identified in some other vaccination studies, even if the vast majority of vaccination programs in Africa have had a tremendous positive public health impact. The efficacy of bacillus Calmette-Guérin vaccination was observed to be lower in African infants than in European ones (52). West Africans showed lower T-cell responses following vaccination with an HIV vaccine candidate than did South Africans and North Americans (7). The efficacy of the licensed yellow fever vaccine 17D was also found to be lower in the African population than the European one; an activated immune environment prior to vaccination was hypothesized (50). In the case of Ebola vaccine, as the protective level has not been determined yet, we do not know if the difference in antibody concentrations has implications on the efficacy of the vaccine. Yet the observed difference in long-term antibody responses between East African sites and the UK site is an interesting outcome that would justify additional mechanistic studies to identify which factors contribute to these differences.

The fact that immune memory is not considered in the model represents a limitation, especially in terms of prediction of the response to exposure to wild-type virus. However, the role of the memory response and the immune response levels required for protection are not known at the moment. Another limitation of the model resides in the assumption that the number of SL and LL cells decreases from 7 days after the boost immunization onwards. Nevertheless, this assumption is supported by several experiments which showed that the peak of the B cell response was reached a few days after immunization (30–32, 48). This assumption could have an influence on the estimated value of the SL ASCs’ half-life, but it does not modify the result that the vaccine regimens can impact the dynamics of the antibody-secreting cells shortly after the boost, with a minor effect on the long term. Moreover, the precision of estimation of the parameters of the model is limited by the low number of subjects (as the data were generated from phase 1 trials), the lack of data on the number of plasmablasts, and the lack of measurements beyond 1 year. However, the statistical analysis using a population approach allowed determination of a lower bound of the long-term response. These results will benefit from additional data coming from phase 2 studies to confirm the robustness of the long-term response. Several studies showed that antibody responses in humans do not reach steady-state levels until approximately 2 to 3 years after infection or vaccination (46). More data should also allow a better identification of the half-lives of the two ASC populations and will increase the statistical power of the analysis. Moreover, the differences between geographic regions will be refined using data from West African subjects. Additional studies looking at the effects of other factors on the immune response, such as malaria coinfection, may help explain these potential differences.

In conclusion, this first modeling study estimates promising binding antibody responses to prime-boost regimens combining Ad26 and MVA in an Ebola vaccine. The antibody concentrations reached 1 year after the prime immunization could be maintained over years thanks to LL ASCs with an estimated half-life of at least 5 years. While long-term antibody persistence was not found to be influenced by the vaccine regimen in the model, the geographic region could potentially impact the long-term antibody concentrations through its effect on dynamic parameters associated with the LL ASCs.

## MATERIALS AND METHODS

### Ethics statement.

The UK trial protocol and study documents were approved by the UK National Research Ethics Service. The Kenya trial protocol and study documents were reviewed and approved by the local Ethics Committee and the Kenyan regulatory authority. The Uganda/Tanzania trial protocol and study documents were reviewed and approved by the Tanzanian Medical Research Coordinating Committee of the National Institute for Medical Research, the Tanzania Food and Drugs Authority, the Uganda Virus Research Institute Research and Ethics Committee, the Uganda National Council for Science and Technology, the Uganda National Drug Regulatory Authority, and the Ethics Committee of the London School of Hygiene and Tropical Medicine. These trials were conducted in accordance with the principles of good clinical practice and the Declaration of Helsinki, and all participants gave formal, written consent before undergoing any trial-related procedure.

### Immunogenicity measurements.

We analyzed data from three randomized, observer-blind, placebo-controlled, phase 1 trials in four countries on healthy volunteers aged 18 to 50. The trials aimed at assessing the safety and tolerability of two novel candidate Ebola vectors combined in different prime-boost regimens. The first vector is a monovalent, recombinant, E1/E3 deletion, replication-defective, adenovirus type 26 vector vaccine encoding Ebola virus Mayinga variant GP (Ad26.ZEBOV). It was produced in PER.C6 human cells and injected in a single dose at a concentration of 1 × 10^11^ viral particles/ml. The second vector is a recombinant, replication-defective, modified vaccinia Ankara vector vaccine (MVA-BN-Filo) expressing Mayinga variant GP, Sudan virus Gulu variant GP, Marburg virus Musoke variant GP, and Tai Forest virus nucleoprotein. It was produced in chicken embryo fibroblasts and injected at a concentration of 2 × 10^8^ median tissue culture infective doses (TCID_50_)/ml.

**FIG 11:**
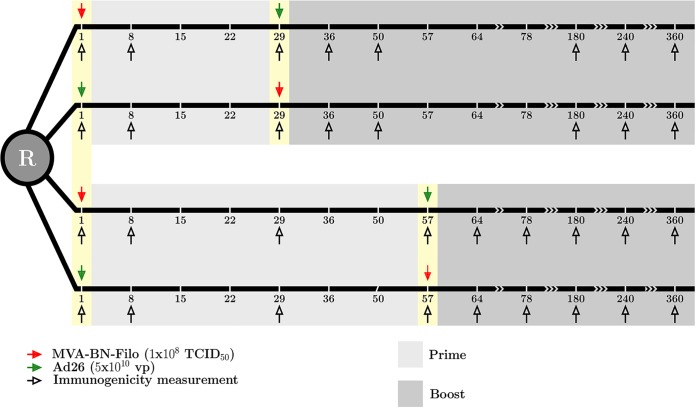
Design of EBOVAC1 trials.

Trials were carried out in the United Kingdom, Kenya, and Uganda/Tanzania. Results of the trials were described previously (see references 2 and 9 for the UK trial, reference 10 for the Kenya trial, and reference 11 for the Uganda/Tanzania trial). Within each trial, eligible participants were equally randomized into four vaccination regimens (within each they received active vaccine or placebo in a 5:1 ratio): two with MVA-BN-Filo as a prime vaccine on day 1 followed by Ad26.ZEBOV on day 29 or day 57 (MVA/Ad26 D29 and MVA/Ad26 D57) and two with a prime immunization of Ad26.ZEBOV at day 1 boosted by MVA-BN-Filo on day 29 or day 57 (Ad26/MVA D29 and Ad26/MVA D57). In the United Kingdom, there was an additional open-label group receiving Ad26.ZEBOV on day 1 followed by MVA-BN-Filo on day 15. This arm was not included in the analysis, as this regimen was not included in East African countries. We included in the analysis only subjects who received both prime and boost immunizations, which corresponded to a total of 177 subjects over all groups and countries. Subjects were followed up to 1 year after receiving the prime immunization, with consecutive immunogenicity assessments performed on blood samples. These samples were taken before prime and boost immunizations, 7 days after prime and boost immunizations and 21 days after the boost immunization. Subjects allocated to groups receiving a boost immunization at day 57 had an additional sample taken at day 29. Further samples were taken at days 180, 240, and 360 after the prime immunization. The design of the trials is summarized in Fig. 11. We analyzed antibody concentrations as the total IgG response against Ebola virus Kikwit variant GP: this was assessed by enzyme-linked immunosorbent assay (ELISA) from BBRC (Battelle) in the UK and Uganda/Tanzania trials and Q2 Solutions in the Kenya trial. Moreover, cellular data obtained from intracellular cytokine staining (ICS) at the HIV Vaccine Trials Network (HVTN) laboratory, Fred Hutchinson Cancer Research Center, on frozen peripheral blood mononuclear cells (PBMC) were explored. The HVTN intracellular cytokine staining (ICS) assay has been validated with HIV and cytomegalovirus (CMV) peptides for IL-2 and IFN-γ analyses (53), and an EBOV GP peptide validation was added as a validation extension by Janssen. PBMC were stimulated with one of two peptide pools covering the GP from Mayinga variant of Ebola virus (Think Peptides, UK). A 16-color staining panel was used, and our analysis was based on the total percentage of CD4^+^ T cells producing IL-2, TNF-α or IFN-γ. Further details of immunogenicity measurements are given in the appendix of reference 2. We focused on the data sampled after the boost immunization in subjects who received both prime and boost immunizations with active components, since we are mainly interested in the duration of the antibody response and its decrease after the observed peak following the boost immunization.

### Mechanistic model.

A preliminary analysis of the decrease of antibody concentrations was performed using linear mixed models. It is described in the appendix. However, our main approach relies on mechanistic models divided in three layers, described in reference 54: first, we used a mathematical model, based on ordinary differential equations and describing the dynamics of the biological process, as was done for hepatitis A vaccine (45). Then we used a statistical model accounting for the interindividual variability and the effects of covariates on the parameters. Finally, we considered an observation model, as immunological measurements do not cover all compartments of the mathematical model.

The mathematical model, represented in Fig. 1, relies on the hypothesis that antibodies are produced by two distinct populations of ASCs, differing by their decay rate (45). It contains three compartments: the SL cells (*S*), the LL cells (*L*), and the antibodies (*Ab*). Time was rescaled in order to consider only the dynamics of antibody concentrations from 7 days after boost immunization, after which both populations of ASCs decrease with time. The corresponding ordinary differential equations are the following:

(1) $$\frac{\mathrm{dS}}{\mathrm{dt}}=-\delta_{S}S$$

(2) $$\frac{\mathrm{dL}}{\mathrm{dt}}=-\delta_{L}L$$

(3) $$\frac{\mathrm{dAb}}{\mathrm{dt}}=\theta_{S}S+\theta_{L}L-\delta_{\mathrm{Ab}}\mathrm{Ab}$$

with δ corresponding to decay rates and θ to production rates. The equation for the antibodies dynamics can be written as

(4) $$\frac{\mathrm{dAb}}{\mathrm{dt}}=\phi_{S}e_{}^{-\delta_{S}t}+\phi_{L}e_{}^{-\delta_{L}t}-\delta_{\mathrm{Ab}}\mathrm{Ab}$$

with φ_S_ = θ*_S_S*_0_ and φ*_L_* = θ*_L_L*_0_, where *S*_0_ = *S* (*t* = 0) and *L*_0_ = *L*(*t* = 0) are the initial conditions at 7 days after the boost immunization. As SL and LL ASC populations were not observed, θ*_S_* and S_0_ could not be identified separately (the same was the case with θ*_L_* and *L*_0_). The initial condition *Ab*(*t* = 0) is given by the data (measured 7 days after the boost immunization). Among the 177 subjects, only 1 did not have a measure of the antibody concentration 7 days after the boost immunization. The value was imputed by using the mean value of his/her group of vaccination in his/her trial, i.e., the mean value of Kenyan subjects in group MVA/Ad26 D29. Finally, we estimated the five following biological parameters: ξ = (φ_S_, δ*_S_*, φ*_L_*, δ*_L_*, δ*_Ab_*).

For the statistical model, as described in reference 54, the parameters *ξ_l_*, *l* = 1.5 are transformed using a logarithm transformation to ensure positivity of production and decay rates. Moreover, a mixed-effect model was introduced on each parameter to account for between-subject variations and possible covariates. Value of parameter ξ̃*_l_* = *ln*(*ξ_l_*) for each subject *i* can be written as follows:

(5) $${{\overset{∼}{\xi }}_{l}}^{i}(t)={{\overset{∼}{\xi }}_{l}}_{0}+\beta_{l}z_{l}^{i}+u_{l}^{i}$$

where ξ̃_*l*_0__ is the intercept and represents the mean ln-transformed value of parameter ξ*_l_* across the population, β*_l_* is a vector of regression coefficients, *z^i^* is a vector of *n_e_* explanatory variables, and *u^i^* is an individual random effect, following a centered normal distribution with variance ω^2^. Random effects were independent from each other and applied on a subset of *q* biological parameters. In practice, after selection (see “Parameter estimation”), we applied random effects on the following parameters: φ*_S_*, φ*_L_*, and δ*_Ab_*. We assessed the effect of *n_e_* = 3 explanatory variables on all parameters except δ*_Ab_*: the order of immunization (binary variable equal to 0 when the subject receives a prime with MVA-BN-Filo boosted by Ad26.ZEBOV and 1 if the subject receives Ad26.ZEBOV and then MVA-BN-Filo), the interval between the two immunizations (binary variable equal to 0 when the subject receives a prime-boost regimen with an interval of 28 days and 1 when the interval is 56 days), and the geographic region (binary variable equal to 0 in Europe and 1 in East Africa). Additionally, we also assessed the effect of the cellular response as an explanatory variable. This was done by considering the percentage of CD4^+^ T cells producing cytokines 7 days after boost immunization. The variable CD4^i^ (boost + 7 days) was added to the vector *z_l_* of explanatory variables, and its effect was estimated on parameter φ*_L_*. Values of β_gr_ and β_CD4_ were estimated as follows:

(6) $${\overset{∼}{\phi }}_{L}^{i\left(t\right)}={\overset{∼}{\phi }}_{L_{0}}+\beta_{\text{gr}}{\text{geographic region}}^{i}+\beta_{\text{CD4}}{\text{CD4}}^{i}\left(\text{boost + 7 days}\right)+u_{l}^{i}$$

with CD4*^i^* (boost + 7 days) the percentage of CD4^+^ T cells producing cytokines 7 days after the boost immunization in participant *i*.

For the observation model, we had access to immunological measurements of IgG binding antibody concentrations against the Kikwit GP in all studies. We assumed that there was a measurement error normally distributed on the log_10_ value of the antibody concentrations. In practice, we assumed we observe for patient i at discrete time *j*:

(7) $$Y\left(t_{\mathrm{ij}}\right)=\log_{10}\left[\mathrm{Ab}\left(t_{\mathrm{ij}}\right)\right]+\varepsilon_{\mathrm{ij}}$$

with

(8) $$\varepsilon_{\mathrm{ij}}∼N\left(0,\sigma_{\mathrm{Ab}}^{2}\right)$$

ε being an additive normally distributed measurement error.

### Parameter estimation.

With the three layers of the mechanistic model, the estimation problem corresponds to the determination of parameter intercepts, regression coefficients, standard deviations of random effects, and standard deviations of measurement errors. The vector of parameters *θ* can be written as follows:

(9) $$\theta =[{\left({{\overset{∼}{\xi }}_{l}}_{0}\right)}_{\text{l}=\text{1.nb}},{\left(\beta_{l}\right)}_{\text{l}=\text{1.ne}},{\left(\omega_{l}\right)}_{\text{l}=\text{1.q}},{\left(\sigma_{l}\right)}_{\text{l}=\text{1.M}}]$$

Estimation was made using NIMROD software, available at http://etudes.isped.u-bordeaux2.fr/BIOSTATISTIQUE/NIMROD/documentation/html/index.html. It uses a maximum likelihood approach (55) with a Newton-like algorithm (56) which approximates the Hessian matrix by using first derivatives of the likelihood. Several criteria ensured the convergence of the algorithm. Moreover, we could account for information on parameters, obtained from biological knowledge and previous estimations in the literature, by adding a prior distribution on these parameters. This led to the determination of the maximum *a posteriori* (MAP) estimator through the maximization of a penalized likelihood (57). In practice, we used a normal prior distribution on the ln-transformed population mean value of biological parameter ξ̃_*l*_0__. Some previous work showed that antibody half-life could vary between a few weeks and a couple of months. Studies of intravenous IgG preparations reported a half-life around 20 to 30 days (33, 34), while studies of passive immunity with maternal transmission of antibodies to infants have reported half-lives varying from 20 days (35) to 35 to 50 days (36–39). These studies also highlighted the interindividual variability over the half-life of antibodies, as well as the possible effect of geographic regions. We used an informative prior distribution on δ̃_*Ab*_0__ such that mean antibody half-life would be 45.2 days, and the variance was chosen such that the 5th to 95th quantiles of the distribution were 6 days to 9 months. Additional sensitivity analyses were performed with a much lower variance on the prior distribution, implying 5th to 95th quantiles of the *a priori* distribution to be 34 to 51 days. We used noninformative prior distributions on parameters ~φ_*S*_0__ and ~φ_*L*_0__, as we did not have any information on their possible value: mean value of the ln-transformed parameters is taken as equal to 0, with standard deviation equal to 10. We used prior distributions on δ̃_*S*_0__ and δ̃_*L*_0__. This helped to constrain the estimation such that δ_*S*_0__ > δ_*L*_0__ as expected by the definition of the SL and LL populations. We used a large prior distribution on δ̃_*S*_0__, as we did not know exactly the time scale of their half-lives. The mean value corresponded to a half-life of 1.88 days, with 5th to 95th quantiles equal to 0.0005 day and 7,000 days. Parameter δ_*L*_ was expected to be close to 0, but as data were collected up to 1 year after the prime immunization, we did not expect the model to be able to distinguish a half-life of more than a few years. To account for this constraint, we used a prior distribution with a mean value corresponding to a half-life of 1.2 year, and 5th to 95th quantiles corresponding to half-lives of 40 days and 14 years. Table 4 sums up the information on the prior normal distributions.

**TABLE 4:** *A priori* distributions of the parameters of the mechanistic model^*a*^

Parameter	Log scale		Natural scale			Mean	Half-life	
	Mean	SD	Mean	Q5	Q95		Q5	Q95
φ*_S_*	0	10	1	7.10^−8^	1 × 10^8^	NA	NA	NA
φ*_L_*	0	10	1	7.10^−8^	1 × 10^8^	NA	NA	NA
δ*_Ab_*	−4.1	1.0	0.017	0.0032	0.086	41 days	8 days	216 days
δ*_S_*	−1.0	5.0	0.37	1 × 10^−4^	1,372	1.88 days	5 × 10^−4^ days	7,029 days
δ*_L_*	−6.5	1.5	0.0015	1 × 10^−4^	0.018	1.3 yrs	40 days	15 yrs

a Q5 and Q95, 5th and 95th quantiles, respectively; NA, not applicable.

Selection of the model random effects and covariates was accomplished by performing estimation on several models that were compared according to two criteria: log likelihood (to be maximized) and approximation of the likelihood based cross-validation criterion (LCVa) (49) (to be minimized). We proceeded in the following way. We first estimated the model parameters using several combinations of two random effects (one on the SL compartment, i.e., either on φ*_S_* or on δ*_S_*, and one on the LL compartment). We selected the best combination and then added a random effect on δ*_Ab_*, which considerably improved the model. The variability on parameter δ*_L_* was complicated to capture: δ*_L_* has an effect mainly on the late dynamics of the antibodies, and data are not available beyond 1 year after the prime immunization. This led us to compare only two combinations of three random effects: on φ*_S_*, φ*_L_*, and δ*_Ab_* and on *δ_S_*, φ_L_, and δ*_Ab_*. Using model criteria, we kept the combination corresponding to the best model, namely, the one with random effects on φ*_S_*, φ*_L_*, and δ*_Ab_*. For the covariate selection, we proceeded with a backward stepwise approach. First, the model was estimated with all covariates (order, interval, and geographic region) on all parameters except δ*_Ab_*. Covariates were removed one by one: in particular, at each iteration *k*, the less significant covariate *Z_k_* was determined using the *P* value of the Wald test and removed. Model criteria ensured that the model was not worse without the covariate *Z_k_* than with *Z_k_*. At the next iteration, the model did not contain covariate *Z_k_*. The least significant covariate *Z_k_*_+1_ was removed in a similar way. These steps were repeated until only significant covariates that could not be removed without altering the performance of the model were kept. Sensitivity analyses were performed: in particular, we estimated first the model with only the geographic region covariate on all parameters and applied the backward stepwise approach. Then we added the order and interval covariates and performed the same approach. Interactions between order and interval were added and tested but were not significant and did not improve the model.

## APPENDIX

Before using the mechanistic model, a preliminary analysis was conducted with linear mixed models to model the decrease of antibodies concentrations from 21 days after the boost immunization (which corresponds to the observed peak). The aim was to estimate linear trends and their variation according to vaccine regimen, geographic region, and clinical characteristics such as age, sex, and body mass index (BMI). Two models were estimated: the single-slope (SS) model and the change-of-slope (CS) model, with a change of slope at time *τ* in order to distinguish the early strong decrease following the peak of antibody to the lighter one at the end of follow-up. Time was rescaled in order to consider only the dynamics of antibody concentrations from 21 days after boost immunization: this time point was redefined as the origin of time. More precisely, for groups receiving a boost at day 29, data were rescaled from day 50 and available measurements were then at days 0, 130, 190, and 310. For groups receiving a boost at day 57, data were rescaled from day 78 with available measurements at days 0, 102, 162, and 282. As two observation points (at least) were needed before and after the value of *τ* to estimate the two slopes in all groups, we chose *τ* = 150 days on the rescaled time. Covariates such as age and BMI were centered around the mean value of the study population. We also used the variable relative to vaccine regimens (order and interval) and geographic settings. The last categorical variable was either the geographic region (=0 for Europe and 1 for East Africa) or the trial (=0 for UK, 1 for Kenya, and 2 for Uganda/Tanzania). Finally, the vector of covariates was:

(A1) Z=(age,sex,BMI,order,interval,geographic setting,order×interval)

We estimated the effect of covariates *Z* on the peak value of antibodies (intercept) and on the decreasing slopes of antibody concentrations. For individual *i* at rescaled time *j*, we write the corresponding antibody concentration *Ab_ij_*. Linear mixed models can be written as:

(A2) $$\text{SS: }\log_{10}\left({\mathrm{Ab}}_{\mathrm{ij}}\right)=\beta_{0}+\gamma_{0i}+\beta_{1}t_{\mathrm{ij}}+\beta_{\mathrm{cov}}^{T}Z_{i}+\beta_{\mathrm{covt}}^{T}Z_{i}t_{\mathrm{ij}}+\varepsilon_{\mathrm{ij}}$$

(A3) $$\begin{array}{l} \text{CS}:\log_{10}\left({\mathrm{Ab}}_{\mathrm{ij}}\right)=\beta_{0}+\gamma_{0i}+\beta_{\mathrm{cov}}^{T}Z_{i}+\beta_{b}t_{\mathrm{ij}}1_{\left\{t_{\mathrm{ij}}<t\right\}}+\beta_{\mathrm{covb}}^{T}Z_{i}t_{\mathrm{ij}}1_{\left\{t_{\mathrm{ij}}<t\right\}}\\ +\beta_{a}t_{\mathrm{ij}}1_{\left\{t_{\mathrm{ij}}\geq t\right\}}+\beta_{\mathrm{cova}}^{T}Z_{i}t_{\mathrm{ij}}1_{\left\{t_{\mathrm{ij}}\geq t\right\}}+\varepsilon_{\mathrm{ij}} \end{array}$$

where 1_{_*_t_*_< τ}_ and 1_{_*_t_*_≥ τ}_ are equal to 1 when *t* < τ and t ≥ τ, respectively, 0 otherwise. In both cases, $\varepsilon_{\text{i}}{}_{\text{j}}∼\text{N}(0,\sigma^{\text{2}})$. We first realized backward selection on the SS model using the geographic region variable. At each step, the covariate with the highest *P* value from the Student test for β (>0.05) was removed from the model. Performance of the models was assessed with the Akaike information criterion (AIC) and Bayesian information criterion (BIC). At the end of the first selection, the CS model was estimated, using only selected variables. At this point, no additional selection was needed. In the final selected model, we also evaluated the trial variable instead of the geographic region. After the selection process, the best SS model was the following:

(A4) $$\begin{array}{l} \log_{10}\left({\mathrm{Ab}}_{\mathrm{ij}}\right)=\beta_{0}+\gamma_{0i}+\beta_{\text{age}}{\text{age}}_{i}+\beta_{\text{order}}{\text{order}}_{i}\\ +\beta_{\mathrm{interval}}{\text{interval}}_{i}+\beta_{\mathrm{gr}}{\text{geographic region}}_{i}\\ +\beta_{1}t_{\mathrm{ij}}+\beta_{{\text{interval}}_{t}}{\text{interval}}_{i}t_{\mathrm{ij}}+\beta_{gr_{t}}{\text{geographic region}}_{i}t_{\mathrm{ij}}+\varepsilon_{\mathrm{ij}} \end{array}$$

The variables age, order, interval, and geographic region have a statistically significant effect on the value of antibody concentration 21 days post boost, and only variables interval and geographic region have a significant effect on the decreasing slope of antibody concentration. Using a CS model significantly improved the BIC criterion (BIC of SS model = 660.5; BIC of CS model = 532.7). However, using the trial variable instead of the geographic region variable improved the AIC criterion but not the BIC one (AIC/BIC of CS model with geographic region variable = 473.8/532.7; AIC/BIC of CS model with trial variable = 463.6/536.0). Table A1 shows the results of the CS linear mixed model using the trial variable. The biphasic decay is well captured by this model, as it can be seen that the decrease is estimated to be stronger before 150 days postboost than after for all groups in all trials. Overall, antibody concentrations had similar values 21 days after boost immunization across countries, with higher values when subjects were boosted at day 57. The decrease was lower in European subjects than in East African ones, both before and after 150 days after boost immunization. It can be noted that the subject characteristics BMI and sex were not statistically associated with antibody concentrations, and age was associated only with the concentration 21 days after boost immunization and not the decrease. Adjusted on other covariates, an increase of 10 years in age induced a reduction of 0.10 log_10_ of antibody concentration at 21 days after boost immunization (confidence interval, −0.17, −0.038), and in the trial population, 50% of the subjects were aged 22 to 35 years. It is clinically less important than the order of vaccine immunizations, as the MVA/Ad26 regimen compared to the Ad26/MVA induces higher concentrations at 21 days after boost immunization of 0.18 log_10_ (confidence interval, 0.086, 0.28) and a boost at day 57 compared to a boost at day 29 induces higher concentrations at 21 days after boost immunization of 0.27 log_10_ (confidence interval, 0.15, 0.38).

**TABLE A1:** Results of the CS linear mixed model

Parameter	Mean value (confidence interval)		
	Europe, UK	East Africa	
		Kenya	Uganda/Tanzania
Antibody concn 21 days postboost (in log10 ELISA units/ml)			
Group MVA/Ad26 D29	3.94 (3.81, 4.07)	3.80 (3.67, 3.93)	3.69 (3.67, 3.93)
Group MVA/Ad26 D57	4.21 (4.08, 4.34)	4.07 (3.94, 4.20)	3.96 (3.94, 4.20)
Group Ad26/MVA D29	3.76 (3.63, 3.89)	3.62 (3.49, 3.75)	3.51 (3.49, 3.75)
Group Ad26/MVA D57	4.03 (3.90, 4.16)	3.89 (3.76, 4.01)	3.78 (3.76, 4.01)
Slope value before 150 days postboost (in log10 ELISA units/ml/30 days)			
Group MVA/Ad26 D29	−0.075 (−0.10, −0.048)	−0.20 (−0.23, −0.17)	−0.17 (−0.19, −0.14)
Group MVA/Ad26 D57	−0.15 (−0.18, −0.12)	−0.28 (−0.31, −0.25)	−0.24 (−0.27, −0.21)
Group Ad26/MVA D29	−0.075 (−0.10, −0.048)	−0.20 (−0.23, −0.17)	−0.17 (−0.19, −0.14)
Group Ad26/MVA D57	−0.15 (−0.18, −0.12)	−0.28 (−0.31, −0.25)	−0.24 (−0.27, −0.21)
Slope value after 150 days postboost (in log10 ELISA units/ml/30 days)			
Group MVA/Ad26 D29	−0.038 (−0.049, −0.027)	−0.12 (−0.13, −0.11)	−0.089 (−0.10, −0.078)
Group MVA/Ad26 D57	−0.086 (−0.098, −0.074)	−0.16 (−0.18, −0.15)	−0.14 (−0.15, −0.12)
Group Ad26/MVA D29	−0.038 (−0.049, −0.027)	−0.12 (−0.13, −0.11)	−0.089 (−0.10, −0.078)
Group Ad26/MVA D57	−0.086 (−0.098, −0.074)	−0.16 (−0.18, −0.15)	−0.14 (−0.15, −0.12)

This preliminary analysis showed the importance of modeling the biphasic decay of antibody concentrations, as a CS model was better than an SS one. Moreover, it highlighted the differences in immune response between East African and European subjects, especially on the decreasing slope of antibody concentrations. Finally, no subject-specific factors had an effect on the dynamics of antibody concentrations except for age, but with a lower impact than geographic region and vaccine-related factors. Only the last factors were considered to potentially affect the dynamics of the humoral immune response in the mechanistic model.

A final check was conducted after parameter φ*_L_* of the mechanistic model was estimated to be significantly different between East African and European subjects. As the proportion of women included in the UK trial is higher than the one in East Africa (64% versus 29% and 20%) and the average age is 10 years higher in the UK trial, as seen in Table 1, the variables age and sex were tested separately as additional covariates on the parameter φ*_L_*, with or without the geographic region variable. Without the geographic region variable, the estimated effect of age and sex was not significant (*P* value = 0.54 and 0.23, respectively). With the geographic variable, the estimated effect of sex was not significant either (*P* value = 0.46) and the effect of age was significant (*P* value = 0.045) but with a low magnitude compared to the effect of geographic region (β = 0.024 for a 10-year difference versus a difference of β = 1.36 between European and East African subjects). These results suggested that the difference of φ*_L_* value between the geographic regions could not be explained by potential confounding demographic factors.
